# Chaperonin–Dendrimer Conjugates for siRNA Delivery

**DOI:** 10.1002/advs.201600046

**Published:** 2016-05-27

**Authors:** Martin G. Nussbaumer, Jason T. Duskey, Martin Rother, Kasper Renggli, Mohamed Chami, Nico Bruns

**Affiliations:** ^1^ Department of ChemistryUniversity of BaselKlingelbergstrasse 804056BaselSwitzerland; ^2^ C‐CINACenter for Cellular Imaging and NanoAnalytics BiozentrumUniversity of BaselMattenstrasse 264058BaselSwitzerland; ^3^ Adolphe Merkle InstituteUniversity of FribourgChemin des Verdiers 41700FribourgSwitzerland

**Keywords:** drug delivery nanosystems, nucleotide delivery vehicle, protein cages, protein–polymer conjugates, RNA interference, small interfering RNA

## Abstract

The group II chaperonin thermosome (THS) is a hollow protein nanoparticle that can encapsulate macromolecular guests. Two large pores grant access to the interior of the protein cage. Poly(amidoamine) (PAMAM) is conjugated into THS to act as an anchor for small interfering RNA (siRNA), allowing to load the THS with therapeutic payload. THS–PAMAM protects siRNA from degradation by RNase A and traffics KIF11 and GAPDH siRNA into U87 cancer cells. By modification of the protein cage with the cell‐penetrating peptide TAT, RNA interference is also induced in PC‐3 cells. THS–PAMAM protein–polymer conjugates are therefore promising siRNA transfection reagents and greatly expand the scope of protein cages in drug delivery applications.

## 1 Introduction

Specific short double‐stranded RNA, the so‐called small interfering RNA (siRNA), induces the enzymatic breakdown of complementary messenger RNA in the cytosol. By interfering with the cellular pathway of disease‐related proteins, siRNA is a promising drug, e.g., against cancer.^1^ Moreover, siRNA has become an important tool for fundamental biological research, as it allows to knockdown gene expression in vitro.[[qv: 1a]] However, some intrinsic problems must be overcome for effective delivery of siRNA into cells, such as the instability of RNA against serum nucleases and rapid clearance of siRNA by the kidney due to its small size (<10 nm).^2^ Moreover, siRNA cannot cross the cell membrane as its backbone is too negatively charged.[[qv: 1c]] Therefore, therapeutic siRNA requires transfection agents that protect oligonucleotides and traffic them into cells. Viral‐,^3^ lipid‐,^4^ or polymer‐based^5^ delivery systems have been reported. However, virus‐like particles are prone to be cleared by virus‐specific antibodies^6^ and lipid‐based siRNA delivery systems are rapidly removed by the liver and spleen.^7^ Polymers and dendrimers used for siRNA delivery often exhibit toxicity and nonspecific cell uptake because they are often highly positively charged.^8^ Such drawbacks of transfection agents are reasons that RNA interference (RNAi) has not reached its full clinical potential. New concepts for siRNA delivery systems are therefore of great interest.[[qv: 1a,c]],^6^, ^7^, ^8^, ^9^

Protein cages are large, hollow, and well‐defined macromolecular structures, which are built from multiple copies of one or more protein subunits.^10^ Their capsule‐like structure in combination with the possibility to precisely modify their exterior surface and their cavity by genetic and chemical means has led to a range of nanotechnological and biomedical applications.^10^, ^11^ Protein cages that have been explored for the delivery of drugs or contrast agents include viral capsids and virus‐like particles,^12^ ferritins,^13^ heat shock proteins,^14^ and chaperonins.^15^ However, most protein cages have small pores that restrict drug delivery applications to small molecules or require therapeutic macromolecules to bind to their outer surface.

**Figure 1:**
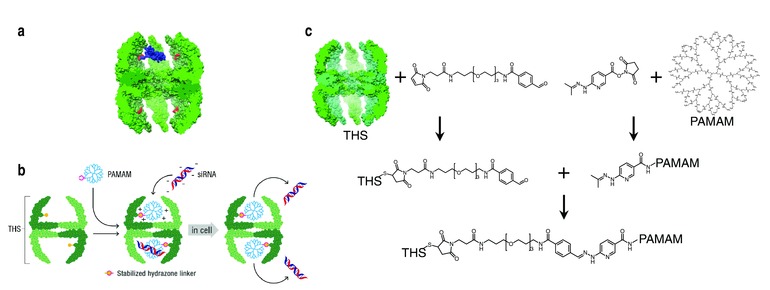
Structure of a THS and schematic depiction of siRNA delivery with a THS–PAMAM conjugate. a) Cut away view of a thermosome (green). Cysteines on the inside surface (red) serve as attachment points for conjugation reactions. To illustrate the proportion of protein cage and dendrimer, a model of PAMAM and the bis‐arylhydrazone linker (blue) was placed into a cavity of THS. b) PAMAM conjugation to the cavity of THS, siRNA binding to THS–PAMAM and transfection of cells by THS–PAMAM conjugate. c) Conjugation strategy to link PAMAM into THS. Cys of THS were modified with the heterobifunctional linker MTFB. PAMAM was modified with the heterobifunctional linker HyNic. After purification of the products from unreacted linkers, THS–MTFB and PAMAM–HyNic were mixed in order to react to THS–PAMAM.

The pores of chaperonins (≈7 nm in diameter) are among the largest found in protein cages and allow macromolecules such as proteins up to 50 kDa[[qv: 11a]],^16^ and polymers[[qv: 11b]] to diffuse in and out of the cavities without affecting the capsule‐like structure of the protein. For this reason, thermosome (THS) was selected as the protein cage to develop a siRNA delivery platform. THS is a group II chaperonin from the archaea *Thermoplasma acidophilum* with a barrel shaped quaternary structure ≈16 nm in diameter. It is composed of 16 alternating α‐ and β‐subunits, which form two hemispheres with a pseudo eightfold symmetry. Each hemisphere encloses a cavity. However, chaperonins do not naturally bind nucleic acids. In order to encapsulate siRNA into THS, the polycationic dendrimer poly(amidoamine) (PAMAM), which can complex siRNA via electrostatic interactions,[[qv: 5d]],^17^ was therefore conjugated into the cavities (**Figure** **1**). The THS–PAMAM conjugate could be loaded with siRNA and protected the oligonucleotide from degradation by RNase. The protein–polymer hybrid was able to deliver siRNA into U87 cells and induced gene silencing. In order to demonstrate the possibility to further equip the protein cage with advanced functionalities, THS–PAMAM was decorated with the cell‐penetrating peptide TAT.^18^ This modification induced uptake and gene silencing in a cell line that otherwise did not respond to THS–PAMAM.

## 2 Results and Discussion

### 2.1 Conjugation of Dendrimer into the Protein Cage

For the conjugation of PAMAM (generation 4, ethylenediamine core) into the cavity of THS, linkers were chosen that form a covalent and stable resonance‐stabilized bis‐arylhydrazone between THS and PAMAM (Figure 1). This linker chemistry has the advantage that the conjugation is stable in a wide pH range and that it can be quantified by UV–Vis spectroscopy.^19^ Cysteines (Cys) in the THS cavities were first reacted with the heterobifunctional linker maleimido trioxa‐6‐formylbenzamide (MTFB).[[qv: 11a]] Their location within the THS is depicted as red residues in Figure 1a. 3.8 ± 0.2 Cys per THS were modified with MTFB, as determined by UV‐Vis spectroscopy (Table S1, Supporting Information). In parallel, PAMAM was modified with a heterobifunctional linker featuring an activated ester and a 6‐hydrazino‐nicotinamide group (HyNic). The degree of modification could be controlled by the concentration of HyNic in the reaction mixture (Figure S1, Supporting Information).

**Figure 2:**
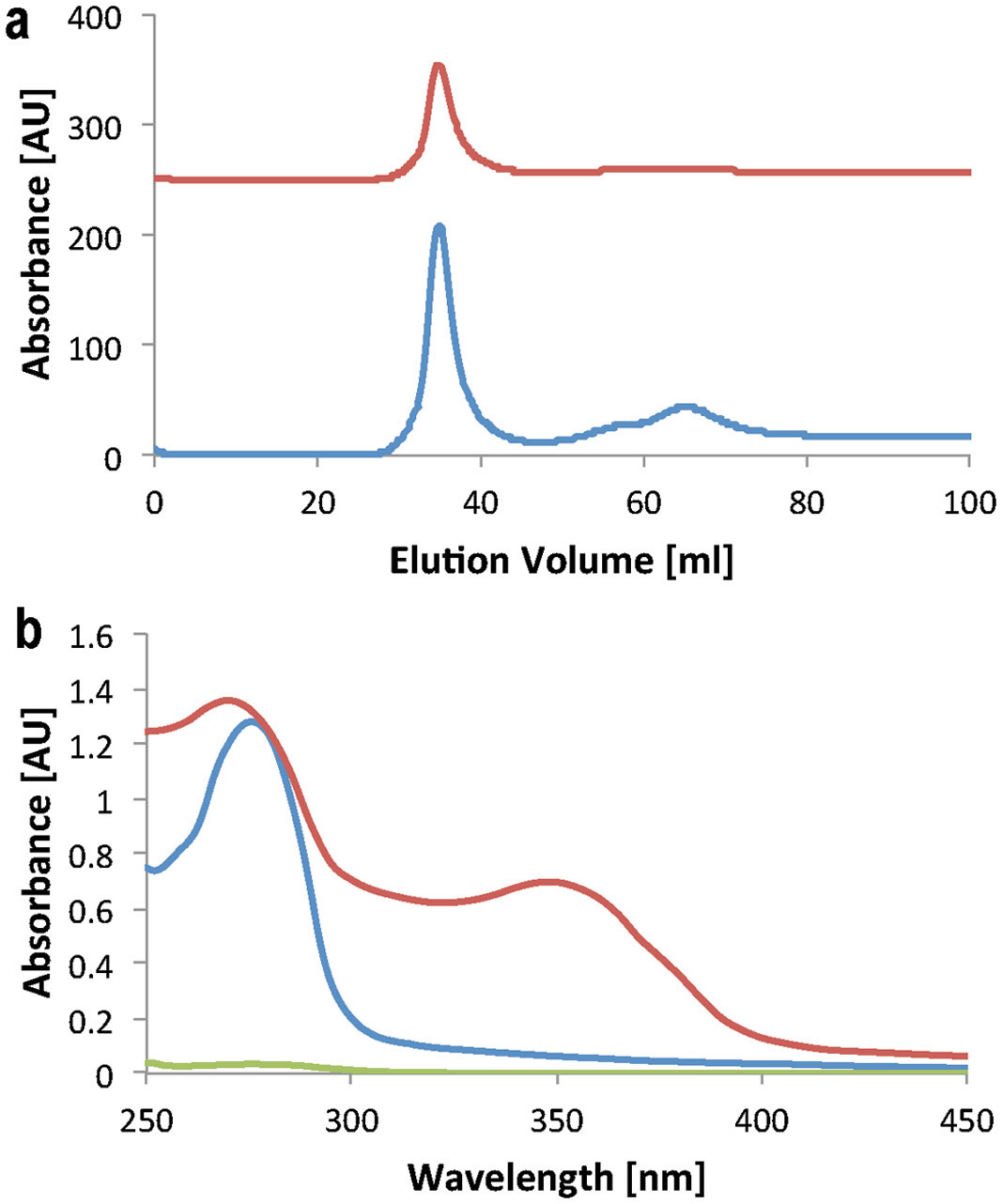
a) Size exclusion chromatogram of the purification of THS–PAMAM from excess of PAMAM, recorded at 280 nm (blue), and 350 nm (red). b) UV–Vis spectra of THS–PAMAM (red; 5.8 × 10^−6^ m), THS (blue; normalized to 5.8 × 10^−6^ m), and PAMAM (green; normalized to 21.3 × 10^−6^ m).

For further experiments, reaction conditions were chosen that resulted in approximately four HyNic groups per PAMAM, which means that about 1/16 of the dendrimer's primary amine groups were modified. In a final step, THS–MTFB was incubated with an excess of PAMAM–HyNic overnight to conjugate PAMAM into the THS. A kinetic study of the conjugation reaction showed that 80% of the THS–PAMAM bonds formed within 3 h (Figure S2, Supporting Information). The protein–polymer conjugate was purified by size exclusion chromatography (**Figure** **2**a). It eluted as a high molecular weight fraction at an elution volume of 35 mL and could be detected simultaneously at 280 nm (resulting from the absorbance of THS–PAMAM) and at 350 nm (resulting from the bis‐arylhydrazone linker). A second fraction at an elution volume of 65 mL absorbed only in the 280 nm channel. It contained the excess of PAMAM that was not bound to THS. The baseline‐separated elution peaks illustrate the successful separation of THS–PAMAM conjugate from free PAMAM. Native polyacrylamide gel electrophoresis (PAGE), transmission electron microscopy (TEM), and cryo‐TEM reveal that the integrity of the protein cage was not affected by the conjugation of PAMAM (Figure S3–S5, Supporting Information). The UV–Vis spectrum of the conjugate showed the absorption of proteins and PAMAM at 280 nm and a peak at 354 nm that is characteristic for the bis‐arylhydrazone linker (Figure 2b). We calculated from such spectra that 3.8 ± 0.2 hydrazone bonds per THS formed, suggesting that all of the modified cysteines in the THS were linked to PAMAM.

**Figure 3:**
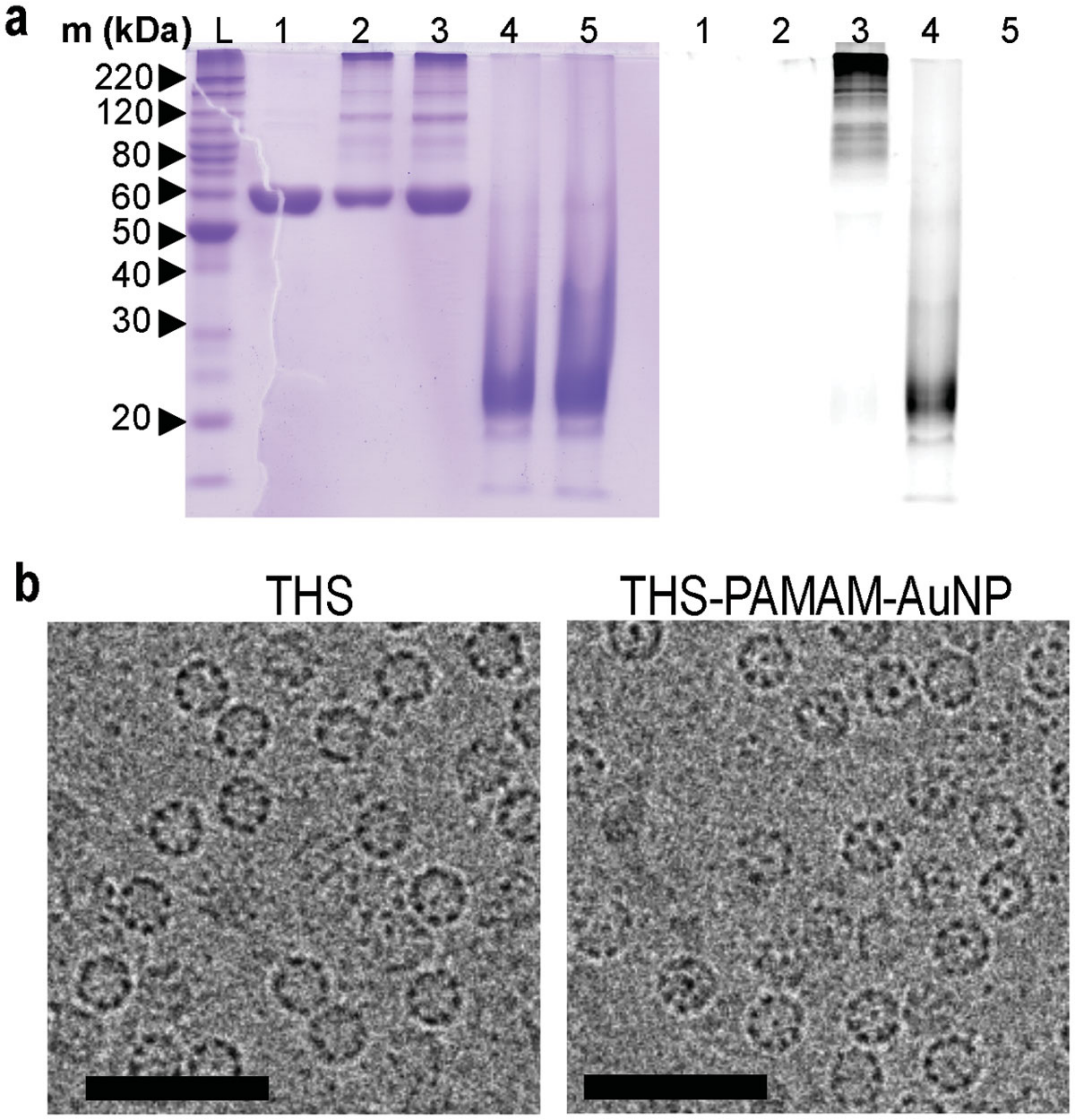
SDS‐PAGE of THS (1), THS–PAMAM (2), THS–PAMAM–Atto647 (3), PAMAM–Atto647 (4) and PAMAM (5), stained with Coomassie Blue (left lanes) and inverted fluorescence image (right lanes). b) Cryo‐TEM images of THS (left) and THS–PAMAM–AuNP (right); scale bars: 50 nm.

The change in molar mass of THS subunits due to PAMAM conjugation was analyzed with SDS–PAGE (**Figure** **3**a). The THS subunits have a molecular weight of ≈58 kDa. PAMAM exhibits a smeared band with a maximum slightly above 20 kDa. THS–PAMAM shows additional smeared lines at higher molecular weight than the THS subunits. In order to distinguish whether these new bands originate from PAMAM or not, the dendrimer was modified with an UV‐active dye (Atto647) and conjugated into the THS. While the THS subunits are not visible in the UV image of the gel, most of the new bands in the conjugate are (Figure 3a). Therefore, it can be concluded that PAMAM is covalently bound to THS subunits. Moreover, the gel shows that there was no free PAMAM in the THS–PAMAM solutions. In order to assess the location of the dendrimer in the conjugates, gold nanoparticles were bound to PAMAM that was then conjugated to THS. Cryo‐TEM revealed gold nanoparticles (AuNP) within THS, confirming that the dendrimer was encapsulated into the protein cage (Figure 3b; Figure S5, Supporting Information).

### 2.2 Binding of siRNA to THS–PAMAM

**Figure 4:**
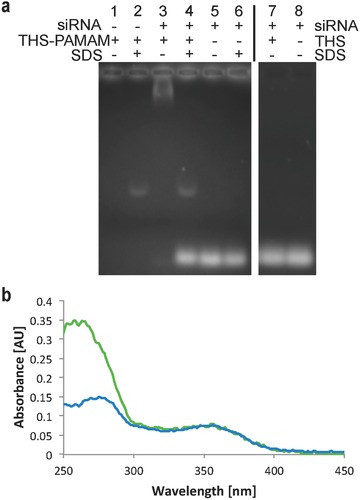
Binding of siRNA by THS–PAMAM. a) EMSA of various combinations of siRNA, THS–PAMAM, and SDS. b) UV–Vis spectrum of THS–PAMAM (green) and THS–PAMAM–siRNA (blue).

Binding of siRNA to THS–PAMAM was demonstrated with an electrophoretic mobility shift assay (EMSA). siRNA that was incubated with THS–PAMAM migrated only a few millimeters in the gel, while free siRNA moved farther (**Figure** **4**a). Incubation of siRNA with THS (i.e., without PAMAM) had no influence on the migration of the nucleic acid. These results indicate that siRNA bound to the PAMAM within THS and that the dendrimer is necessary to convert THS into a nucleotide delivery vehicle. The reversible character of this complexation was demonstrated by the addition of SDS.^20^ THS–PAMAM released siRNA that migrated as fast as free siRNA in the electrophoresis gel (Figure 4a). The additional band in sample 2 and 4 does not originate from a siRNA complex but results from an interaction between SDS and THS–PAMAM.

After rigorous separation of free siRNA from THS–PAMAM, the amount of siRNA bound to THS–PAMAM was measured by UV–Vis spectroscopy (Figure 4b). The presence of siRNA caused an increase of absorbance at 260 nm, which is the characteristic absorbance band of nucleic acids. These spectra allowed calculating that on average one siRNA was captured by each THS–PAMAM.

The morphology of complexes between siRNA and THS–PAMAM was characterized with TEM and dynamic light scattering (DLS) (Figure S6, Supporting Information). TEM images show that the protein cage–polymer conjugate maintained its typical spherical structure upon binding of siRNA. Some protein nanoparticles appear to have aggregated. This observation was confirmed by DLS, which measured an average particle hydrodynamic diameter of 98.0 ± 18.3 nm. In contrast, a hydrodynamic diameter of 14.6 ± 2.2 nm was measured for THS–PAMAM without siRNA. Most likely, some siRNA bound to more than one cationic dendrimer and therefore connected THS–PAMAM particles.

### 2.3 Protection of siRNA from Degradation

**Figure 5:**
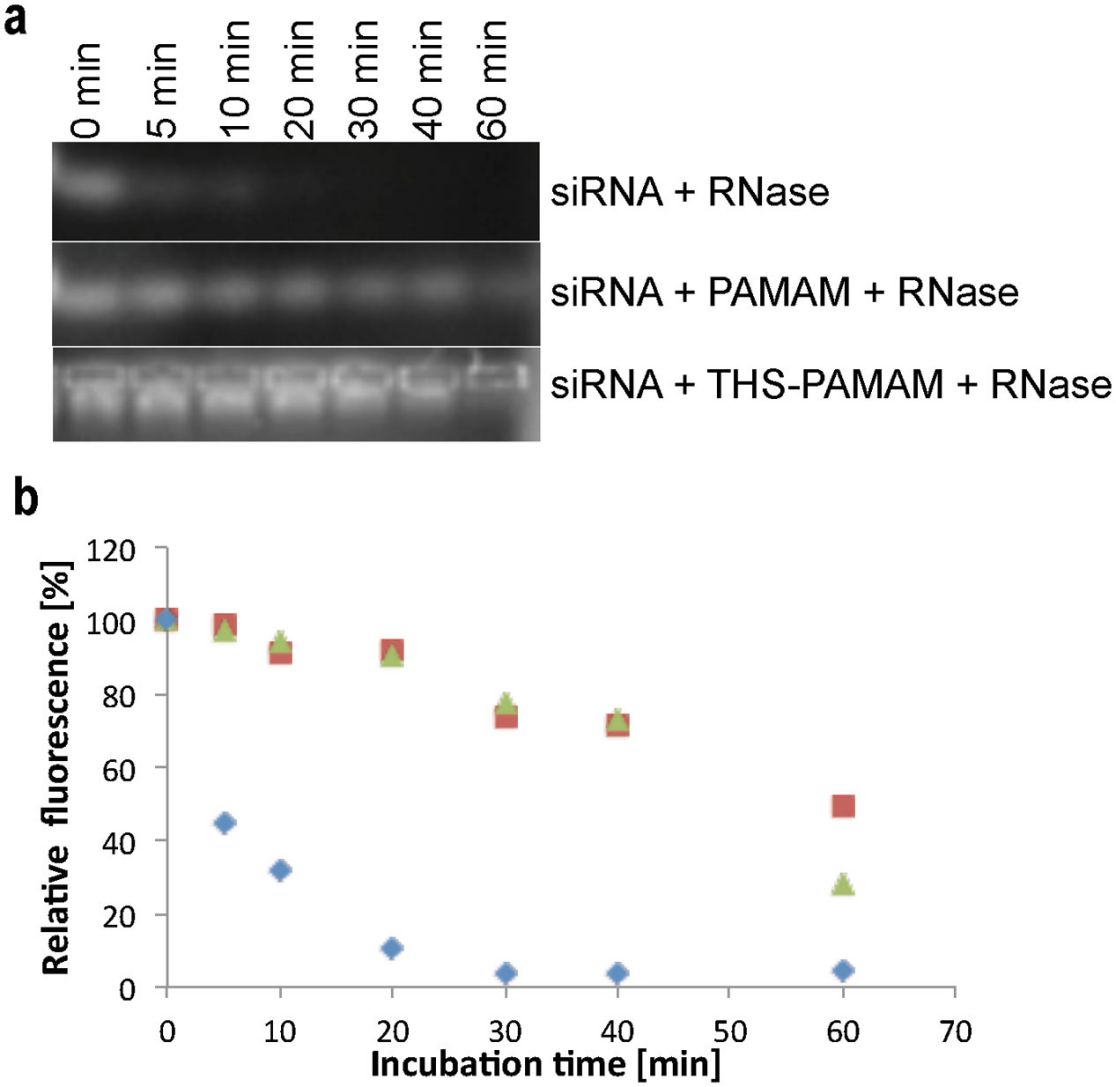
Stabilization of siRNA by THS–PAMAM and PAMAM against enzymatic degradation by RNase A. a) Digestion experiment of siRNA, PAMAM‐siRNA and THS–PAMAM–siRNA monitored by agarose gel electrophoresis. b) Quantification of fluorescence, which corresponds to the concentration of intact siRNA, in the digestion experiments: siRNA (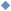), PAMAM–siRNA (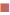) and THS–PAMAM siRNA (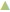).

For siRNA delivery, it is crucial to protect siRNA from degradation by nucleases, as these enzymes are ubiquitously present in extracellular fluids.^8^ Free siRNA was degraded by RNase A to 50% of its original concentration in less than 5 min, whereas it took more than 40 min to degrade siRNA bound to THS–PAMAM to 50% (**Figure** **5**). Similar results were found with siRNA complexed by PAMAM. Therefore, the protein–dendrimer conjugate stabilized siRNA against enzymatic degradation with a similar efficiency than the established dendritic delivery agent.[[qv: 5d]]

### 2.4 Cell Uptake of THS

**Figure 6:**
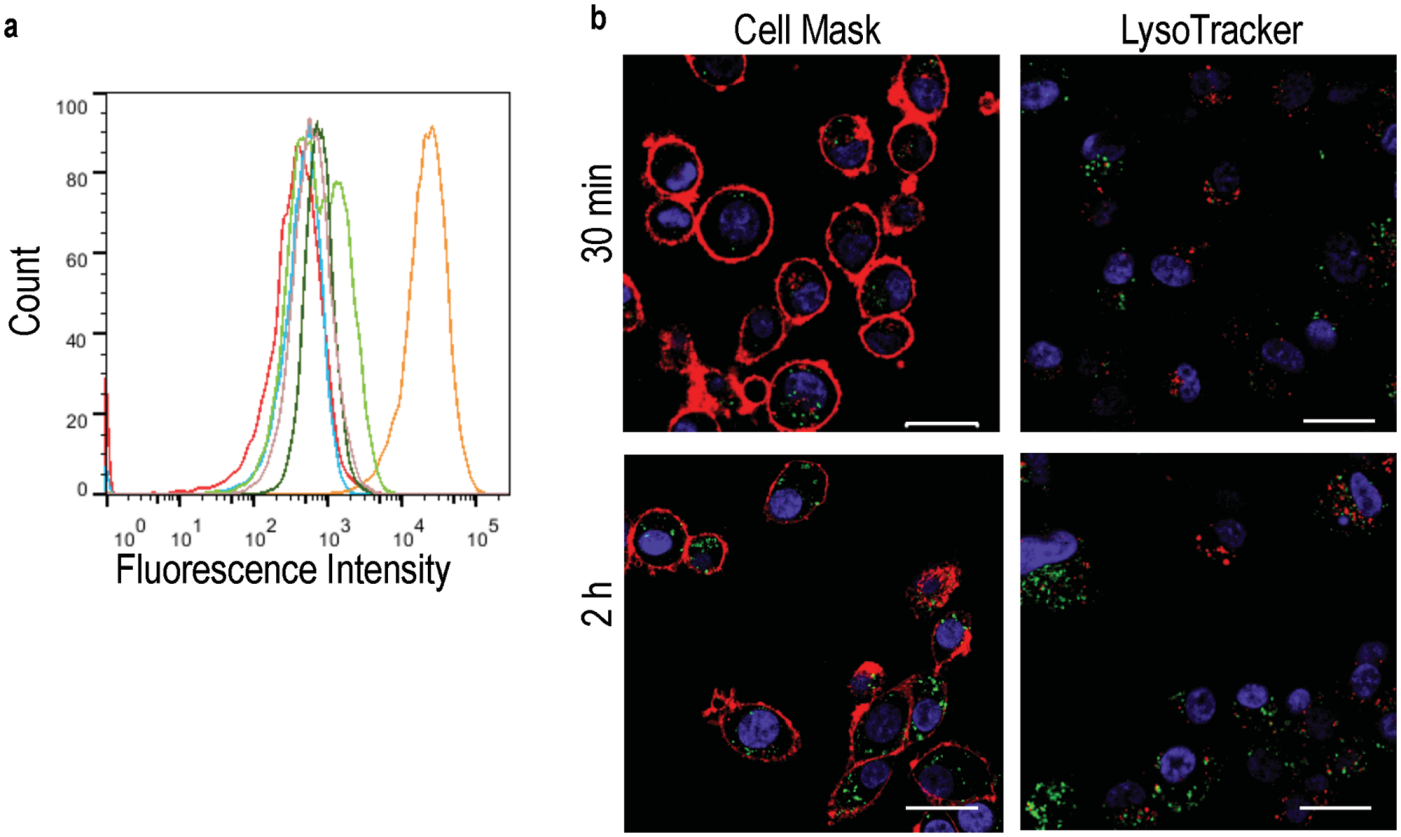
Cell uptake of THS. a) Flow cytometry of different cell lines incubated with THS–Atto647: MCF‐7 (red), PC‐3 (brown), HUVEC (light green), CHO‐K1 (dark green), U87 (orange) and as a control U87 in the absence of THS–Atto647 (blue). b) Confocal microscopy images of U87 cells incubated with THS–Atto647 for 30 min and 2 h: THS–Atto647 (green), nucleus (blue), left: cell membrane (red), right: acidic compartments (red); scale bars: 20 μm.

Several cell‐lines (PC‐3, HeLa, MCF‐7, CHO‐K1, HUVEC & U87) were tested for the uptake of dye‐labeled THS (THS‐Atto647) with flow cytometry (FACS) (**Figure** **6**a, Table S2, Supporting Information). Only U87 interacted strongly with the chaperonin, possibly because different receptors induce internalization.[[qv: 11f]] The results suggest that U87 cells are suitable targets for siRNA delivery by the protein cage. The interaction of THS–Atto647 with U87 cells was further analyzed with a confocal laser scanning microscope (CLSM) to locate THS–Atto647 in or on the cells (Figure 6b). After incubating the cells for 30 min in a solution of THS–Atto647, the protein cage was taken up into the cytoplasm of the cells. It did not bind to the cell membrane and it was not translocated to the nucleus. Additionally, the cells were treated with the dye LysoTracker that stains acidic cell compartments. This allows to assess if THS–Atto647 accumulates, e.g., in lysosomes. Most of THS–Atto647 was not co‐localized with LysoTracker, allowing to conclude that the uptake either occurs via nonacidic compartments or that THS rapidly escapes from endosomes. The intracellular location of THS‐Atto647 did not change at longer incubation time of 2 h (Figure 6b). Control experiments in which U87 cells were incubated with free Atto647 showed some uptake into the cells, but most of the dye was co‐localized with the cell membrane. Moreover, dye agglomeration occurred (Figure S7, Supporting Information). Taken together, these results prove that the protein cage entered the cells.

**Figure 7:**
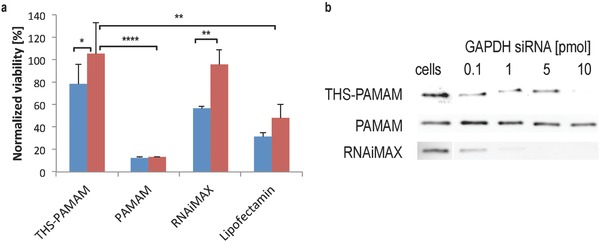
siRNA delivery into U87 cells. a) Normalized viability of U87 cells transfected with KIF11 siRNA (blue) or scrambled siRNA (red) with either THS–PAMAM, PAMAM, Lipofectamine RNAiMAX or Lipofectamine 2000 (10 pmol siRNA per well in all cases). (Two sided t‐test; THS–PAMAM *n* = 12 each; PAMAM, Lipofectamine RNAiMAX, and Lipofectamine *n* = 4 each; *: *p* < 0.05; **: *p* < 0.01; ****: *p* < 0.0001). b) Representative western blots of GAPDH expression in U87 cells transfected with GAPDH siRNA with either THS–PAMAM, PAMAM, or Lipofectamine RNAiMAX.

As THS was rapidly taken up by U87 cells, it was tested if THS–PAMAM can deliver siRNA into the cytosol and trigger gene silencing. To this end, the protein–dendrimer conjugate was loaded with a siRNA that interferes with the messenger RNA of KIF11, a protein of the kinesin family. A knockdown of KIF11 by siRNA induces stop of proliferation, and nonapoptotic, lysosomal cell death.^21^ KIF11 siRNA complexed with THS–PAMAM was added to U87 cells (10 pmol per well siRNA) and the cell viability was measured after 72 h. As a negative control, THS–PAMAM was used to deliver scrambled siRNA (10 pmol per well) to these cells. THS–PAMAM/KIF11 siRNA reduced the viability of the cells to 79% ± 17% normalized to non‐treated cells. THS–PAMAM with scrambled siRNA (**Figure** **7**a) did not affect the viability (106% ± 28%). Thus, THS–PAMAM/KIF11 siRNA resulted in a reduction of U87 cell viability by 25% when compared with THS–PAMAM with scrambled siRNA (Figure 7a). Even though the error bars of the two sets of measurements overlap to some extent, the difference between KIF11 siRNA and scrambled siRNA is significant (significance level in a two‐sided Student's t‐test: *p* = 0.029). These transfection results are comparable to other studies on KIF11 siRNA delivery, e.g., using lipid‐coated poly(lactic‐*co*‐glycolic acid) nanoparticles as transfection agent.^22^ In contrast, PAMAM without THS was highly cytotoxic and reduced the cell viability to 13% ± 1%, independent of the presence of siRNA or the type of siRNA (Figure 7a and Figure S8, Supporting Information). We used the same concentration of PAMAM (0.38 × 10^−6^ m) as in the test with the THS–PAMAM conjugates in order to allow for a direct comparison of the two sets of experiments. These results show that PAMAM alone cannot be used as siRNA delivery reagent at these conditions and stresses the importance of encapsulating PAMAM into the protein cage, thereby shielding the positive charges of PAMAM which are the source of the dendrimer's toxicity.

To set these findings into perspective, U87 cells were transfected with KIF11 siRNA and scrambled siRNA using Lipofectamine RNAiMAX (RNAiMAX) and Lipofectamine 2000 (LF), two standard reagents for in vitro delivery of nucleic acids (Figure 7a).^23^ KIF11 siRNA transfected with RNAiMAX and LF decreased the cell viability to 57% ± 2% and 32% ± 4%, respectively, when normalized to nontreated cells. Viability of the cells stayed at 96% ± 14% when RNAiMAX and scrambled siRNA was used, indicating that this transfection agent was not toxic. However, LF with scrambled siRNA reduced cell viability significantly (*p* = 0.0063) to 48% ± 13%. Similar results were obtained with LF in the absence of RNA (Figure S8, Supporting Information). Thus, the pronounced reduction in cell viability observed for siRNA‐loaded LF was mainly due to the toxicity of the transfection agent, and only partially caused by KIF11 silencing. Taking this into account, the transfection efficiency of THS–PAMAM for KIF11 siRNA was slightly lower than the efficiency of LF and about two thirds of the efficiency of RNAiMAX. However, LF exhibited unspecific toxicity against the investigated cell line, while THS–PAMAM was not cytotoxic.

To further evaluate the effectiveness of siRNA delivery by THS–PAMAM, the knockdown of another gene was investigated. Glyceraldehyde 3‐phosphate dehydrogenase (GAPDH) is a marker protein commonly used in such studies.^24^ Therefore, siRNA against GAPDH was transfected with THS–PAMAM into U87 cells (Figure 7b). The protein–polymer conjugate efficiently silenced GAPDH expression at 10 pmol per well siRNA. In comparison, PAMAM had only minor effects on the GAPDH level, even when 10 pmol per well of siRNA was transfected with the dendrimer. Therefore, PAMAM alone has only very weak transfection efficacy, but encapsulated in THS, it becomes a potent siRNA transfection agent. RNAiMAX was even more efficient and showed silencing already at 0.1 pmol per well siRNA. However, the gold standard for in vitro siRNA delivery has its drawbacks, for example it cannot be further functionalized on its surface and it is not suitable for in vivo experiments.[[qv: 1b]]

### 2.5 Decoration of THS–PAMAM with a Cell‐Penetrating Peptide

In contrast to the two commercial transfection reagents, THS–PAMAM can be further modified on its surface. To show this versatility of the protein cage and to perform siRNA‐induced gene silencing in another cell type, THS–PAMAM was decorated with the cell‐penetrating peptide TAT, which is derived from the transactivator of transcription (TAT) of human immunodeficiency virus (HIV).^25^ The peptide is known to facilitate the internalization of drugs, proteins, and nanoparticles.^18^ TAT was conjugated to the lysines of THS–PAMAM with a bis‐arylhydrazone linker (Figure S9, Supporting Information). An increase in molar mass of the THS subunits due to the conjugation of TAT was observed in SDS–PAGE (Figure S10a, Supporting Information). On average, 6.7 ± 0.2 TAT peptides were conjugated to each THS–PAMAM, as determined from the UV absorbance band at 354 nm (Figure S10b, Supporting Information).

**Figure 8:**
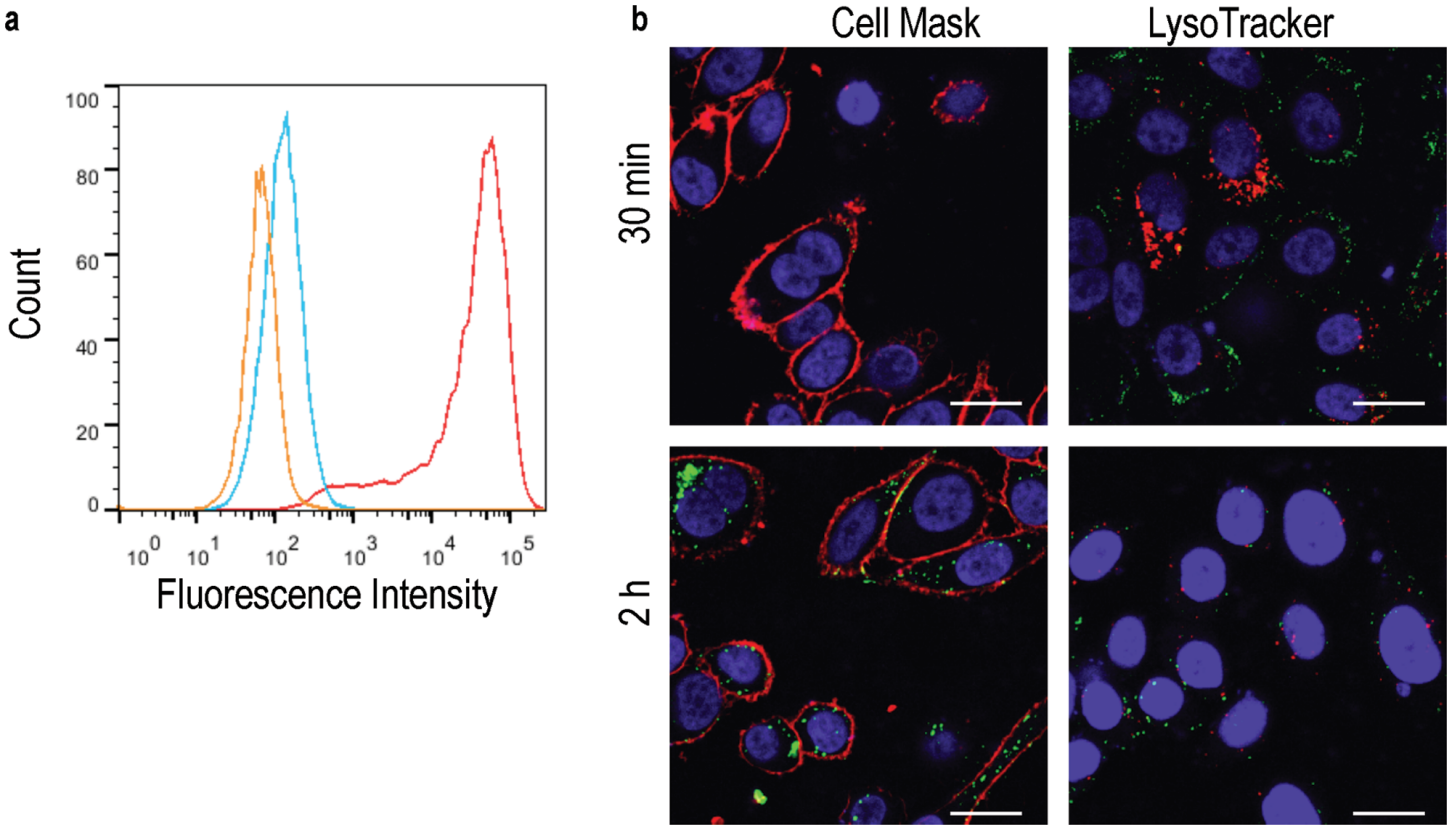
Uptake of TAT‐THS by PC3 cells. a) Flow cytometry of PC‐3 (orange), PC‐3 incubated with TAT–THS–Atto647 (red) and PC‐3 incubated with THS‐Atto647 (blue). b) confocal microscopy images of PC‐3 cells incubated with TAT‐THS–Atto647 for 30 min and 2 h; TAT–THS–Atto647 (green), nucleus (blue), left: cell membrane (red), right: acidic compartments (red); scale bars: 20 μm.

TAT is known to facilitate uptake of cargo into PC‐3 cells.^26^ Therefore, these cells were incubated with a fluorescently labeled, TAT‐decorated THS, and analyzed by flow cytometry (**Figure** **8**a). The median fluorescence of the cells with TAT–THS–Atto647 increased about 630 times compared with the background fluorescence of PC‐3 cells. In comparison, unmodified THS–Atto647 showed only minor uptake by PC‐3 cells, which manifested itself in an increased fluorescence of a factor of three. The interaction of TAT–THS–Atto647 with PC‐3 cells was investigated by confocal microscopy (Figure 8b). After 30 min of incubation, TAT–THS–Atto647 was located at the cell membrane of PC‐3 cells and had not yet entered the cells. After 2 h of incubation, TAT–THS–Atto647 was internalized into cells. The protein cage was not co‐localized with LysoTracker, i.e., with acidic compartments. As expected, it was also not located at the cell nucleus. Concluding, the cell‐penetrating peptide is able to traffic the protein cage into these cells. After a phase where TAT–THS–Atto647 is located at the cell membrane, the protein cage is translocated to the cytosol. These findings suggest that the uptake of TAT–THS–Atto647 by PC‐3 and of THS–Atto647 by U87 cells follow different pathways. Taking into account that the general uptake mechanisms of TAT and its diverse cargo are still under scientific debate,^27^ it cannot be concluded whether TAT–THS enters the cells through the endosomal pathway and escapes the endosome, or if it reaches the cytosol by macropinocytosis.^28^ Nevertheless, these results confirm that TAT can translocate relative large proteins into cells that would otherwise not internalize these proteins.^29^

**Figure 9:**
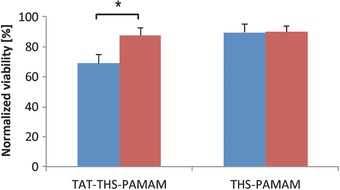
siRNA delivery into PC‐3 cells. Normalized viability of PC‐3 cells transfected with KIF11 siRNA (blue) or scrambled siRNA (red) with either TAT–THS–PAMAM or THS–PAMAM (10 pmol siRNA per well in all cases). (Two sided t‐test; TAT–THS–PAMAM *n* = 4 each; THS–PAMAM *n* = 4 each; *: *p* < 0.05).

TAT‐modified THS–PAMAM was used to deliver siRNA into PC‐3 cells. To this end, TAT–THS–PAMAM was loaded with 10 pmol per well KIF11 siRNA and scrambled siRNA, respectively, and added to PC‐3 cells. As control, the same experiments were performed with THS–PAMAM that lacked the cell penetrating peptide. The viability of cells incubated with KIF11 siRNA‐loaded TAT–THS–PAMAM dropped to 69% ± 6% compared with nontreated cells and to 79% ± 8% (*p* = 0.014) when compared with the cells that were treated with TAT–THS–PAMAM carrying scrambled siRNA (**Figure** **9**). In contrast, the cell viability of THS–PAMAM without cell penetrating peptide enclosing KIF11 siRNA (88% ± 5%) or scrambled siRNA (90% ± 4%) varied only minimally. These results show that TAT‐modified THS–PAMAM is able to transfect siRNA into PC‐3 cells and that the presence of the cell‐penetrating peptide is crucial for this purpose. The performance of TAT–THS–PAMAM in delivering KIF11 siRNA into PC‐3 cells is roughly the same as THS–PAMAM delivering siRNA into U87 cells.

## 3 Conclusion

siRNA is a promising drug as it can be used to silence the expression of disease‐related proteins. Moreover, it is a powerful tool for in vitro molecular biology studies. However, transfection reagents have to be used to traffic the oligonucleotide into cells. We present a delivery platform for siRNA based on the protein cage THS. PAMAM was covalently bound into the THS in order to equip the protein with the ability to bind and release nucleic acids. THS–PAMAM stabilized siRNA against degradation by RNase. THS was taken up by U87 cells but not by PC‐3, HeLa, MCF‐7, CHO‐K1 or HUVEC cells. Significant siRNA‐induced inhibition of proliferation and silencing of GAPDH in U87 was achieved with THS–PAMAM, whereas PAMAM alone was highly toxic to the cells and not efficient in delivering siRNA. Additional modification of THS with the cell‐penetrating peptide TAT allowed for uptake and siRNA delivery into cells that otherwise do not internalize the protein cage.

THS–PAMAM conjugates offer several advantages over free PAMAM as a transfection agent. PAMAM is shielded within the protein cage. The dendrimer can otherwise directly interact with cells, which causes cytotoxicity and initiates uncontrolled uptake.^30^ Decoration of THS with TAT is an example for the versatility of the THS delivery platform. It shows that the protein cage can be easily modified with functional ligands, which will allow engineering it further into a targeted drug delivery system.

The properties of the protein–polymer conjugate to bind, protect and release guests make it an interesting delivery platform with applications well beyond the delivery of siRNA. For example, PAMAM inside the THS could be used to bind a high number of small drugs or MRI‐contrast agents. Moreover, the THS could be used to deliver other therapeutic macromolecules or oligomers, such as pharmaceutically active peptides and small proteins. In conclusion, THS–PAMAM represents a promising and versatile delivery platform for siRNA and has considerable potential as nontoxic nanodelivery system for therapeutic payloads.

## 4 Experimental Section

*Materials*: The THS mutant was expressed and purified as previously reported by Bruns et al.[[qv: 11a,b]] All chemicals were purchased from Sigma‐Aldrich, unless stated otherwise. Maleimido trioxa‐6‐formylbenzamide (MTFB), succinimidyl‐6‐hydrazinonicotinamide acetone hydrazone (HyNic), tetra ethylene glycol pentafluorophenyl ester 4‐formylbenzamide (PEG_4_‐PFB), and 6‐hydrazinonicotinamide acetone hydrazone‐GRKKRRQRRRPPQ‐NH_2_ (TAT–HyNic) were purchased from Solulink (USA). Following cell lines were purchased from ATCC (USA): breast adenocarcinoma cells MCF‐7 (ATCC HTB‐22), Chinese hamster ovary cells CHO‐K1 (ATCC CCL‐61), vascular endothelium cells HUVEC (ATCC CRL‐1730), glyoblastoma cells U‐87 MG (ATCC HTB‐14) and cervical cancer cells HeLa (ATCC CCL‐2). Prostate cancer cells PC‐3 (ATCC CRL‐1435) were a kind gift of Prof. T. Mindt's group (University Hospital Basel) and purchased from HPA Culture Collections (UK). Centrifugal filters were purchased from Merck Millipore (USA), RNase A from Roche (Switzerland), Atto dyes from ATTO‐TEC (Germany), GelRed from Biotium (USA), cell culture flasks and 24‐ and 96‐well plates from BD Bioscience (USA) and 8‐well microscope chamber slides from Nunc (USA). 6X MassRuler DNA Loading Dye, Hoechst 33342, CellMask orange, LysoTracker Red DND‐99, penicillin–streptomycin mix, 100× nonessential amino acids solution, silencer KIF11 (Eg5) siRNA (sequence not revealed by the supplier), Lipofectamine 2000 (LF), and Lipofectamine RNAiMAX, were purchased from Life Technologies (USA). Scrambled siRNA with the sequence 5′‐AGG UAG UGU AAU CGC CUU GTT‐3′ was obtained from Microsynth (Switzerland). DMEM, opti‐MEM, the Micro BCA protein assay kit, the super signal west pico chemiluminescent substrate and Silencer select positive control GAPDH siRNA as well as nuclease free PBS buffer (10×) and nuclease‐free water were purchased from Thermo Scientific (USA). 1.4 nm Monomaleimido nanogold (AuNP‐mal) and 1.4 nm mono‐sulfo‐NHS nanogold (AuNP–NHS) were obtained from Nanoprobes, USA.

*Methods*: All concentrations are given as end concentrations in the reaction mixture. Concentration measurements were performed with NanoDrop 2000c (Thermo Scientific, USA), if not stated otherwise, and calculated with following extinction coefficients: THS ε_280nm_ = 210 880 M^−1^ cm^−1^,[[qv: 11a]] hydrazone bond ε_354nm_ = 29 000 M^−1^ cm^−1^ (according to the manufacturers protocol; Solulink, USA),[[qv: 19a]] siRNA ε_260nm_ = 300 000 M^−1^ cm^−1^ (determined by absorption measurements at different concentrations). Structures of proteins were rendered with the software USCF Chimera V1.10.1.^31^ As there is no structural data set of the open conformation of THS available in the databases, the cryo‐EM density map of the structural similar thermosome Mm‐cpn in its open conformation is shown.^32^ The location of cysteine on the inner surface of the beta subunits was determined by sequence alignment between the THS mutant and Mm‐cpn using ClustalW2 (http://www.ebi.ac.uk/Tools/msa/clustalw2/). A model of PAMAM (modified with the bisaryl hydrazone linker that results from the reaction of HyNic and MTFB) was computed using the software ChemBio3D Ultra 14 by running a MM2 molecular dynamics simulation to relax the molecule, followed by MM2 energy minimization. The resulting 3D model was placed into Mm‐cpn using Chimera.

*SDS‐PAGE*: SDS‐PAGE gels were prepared according to Laemmli's protocol.^33^ Subsequently to denaturing the samples at 95 °C for 3 min the samples were loaded on 12% SDS–PAGE gel and run for 70 min at constant voltage of 200 V. A fluorescence image (Bio‐Rad Gel doc EZ imager) was recorded before Coomassie blue staining.

*Native‐PAGE*: The samples were mixed with 5× loading buffer (150 × 10^−3^ m Tris/HCl pH 6.8, 70% glycerol, 0.01% w/v bromophenol blue), loaded on a 4%–20% precast gradient gel (10 well, mini‐protean, Bio‐Rad, USA) and run in Tris‐glycine buffer (25 × 10^−3^ m Tris pH 8.25, 192 × 10^−3^ m glycine) for 3.5 h at constant 100 V.

*Transmission Electron Microscopy*: For Figure S4 (Supporting Information) 5 μL of 20 × 10^−9^ m THS–PAMAM solution was deposited for 60 s on a glow‐discharged grid. For Figure S6 (Supporting Information), 0.1 × 10^−6^ m THS–PAMAM and 0.1 × 10^−6^ m siRNA was incubated for 10 min at RT and afterward 100× diluted with PBS pH 7.4 (1 × 10^−9^ m THS–PAMAM and 1 × 10^−9^ m siRNA). The dilution step was necessary to identify aggregates; else the THS–PAMAM would be too densely packed to do so. The solutions were then deposited for 60 s on a glow‐discharged grid, washed twice with ddH_2_O and negatively stained twice with 1% uranyl acetate solution. The sample was imaged with a Fei Morgagni 268 D TEM at an accelerating voltage of 80 kV.

*Cryo‐Transmission Electron Microscopy*: A 4 μL aliquot of sample was adsorbed onto glow‐discharged holey carbon‐coated grid (Quantifoil, Germany), blotted with Whatman filter paper and vitrified into liquid ethane at −178 °C using a vitrobot (FEI company, Netherlands). Frozen grids were transferred onto a Philips CM200‐FEG electron microscope using a Gatan 626 cryo‐holder (GATAN, USA). Electron micrographs were recorded at an accelerating voltage of 200 kV and a nominal magnification of 50000×, using a low‐dose system (10 e^−^per Å^2^) and keeping the sample at −175 °C. Defocus values were −4 μm. Micrographs were recorded at 4K × 4K CMOS camera (TVIPS, Germany).

*Electrophoretic Mobility Shift Assays*:^34^ Samples were run for 40 min at constant voltage of 100 V on a 1.2% agarose gel in TRIS‐acetate‐EDTA (TAE) buffer. The gel was stained with GelRed stain and a UV‐image was recorded (Bio‐Rad Gel doc EZ imager). THS–PAMAM concentration of 6 × 10^−6^ m and siRNA concentration of 2 × 10^−6^ m were used for the samples. The samples were incubated for 10 min at RT. To selected samples a final concentration of 0.4% SDS was added and incubated for another 10 min at RT. 10 μL of each sample was mixed with 2 μL loading buffer (6× MassRuler DNA Loading Dye) and loaded on the gel.

*Digestion Assays*: siRNA (2.8 μg, 2 × 10^−6^ m) was incubated with either PAMAM (8.53 μg, 6 × 10^−6^ m), 6 × 10^−6^ m THS–PAMAM or on its own for 10 min at 37 °C in 100 × 10^−3^ m phosphate buffer pH 7.5 with 150 × 10^−3^ m NaCl. Then, 50 U mL^−1^ RNase A was added and the solutions were incubated at 37 °C for defined time intervals (0 min, 5 min, 10 min, 20 min, 30 min, 40 min, and 60 min). SDS was added to a final concentration of 0.4% to specified samples. Immediately thereafter, the samples were analyzed by agarose gel electrophoresis and imaged with a fluorescence gel reader. Fluorescence intensity analysis was performed with the software ImageJ 1.48v (NIH, USA), whereas the fluorescence was normalized to the fluorescence of the samples that were incubated for 0 min.

*THS–PAMAM Conjugation*: THS (1.14 mg, 10 × 10^−6^ m) was reacted with MTFB (121 μg, 2 × 10^−3^ m; 200 equiv. to THS; 12.5 equiv. to subunits) in buffer A (100 × 10^−3^ m sodium phosphate pH 7.5, 150 × 10^−3^ m NaCl) for 2.5 h at RT under shaking. Subsequently, the buffer was exchanged to buffer B (100 × 10^−3^ m sodium phosphate pH 6.5, 150 × 10^−3^ m NaCl). PAMAM (0.85 mg, 500 × 10^−6^ m) was modified in buffer B at RT for 2 h under shaking with a tenfold excess of HyNic (174 μg, 5 × 10^−3^ m). After purification with centrifugal filters, modified THS and modified PAMAM were mixed to a final concentration of 10 × 10^−6^ m (0.95 mg) and 500 × 10^−6^ m (0.71 mg), respectively. The solution was incubated overnight in buffer B at RT under shaking. A kinetic study of the conjugation reaction showed that 80% of the THS–PAMAM bonds formed within 3 h (Figure S2, Supporting Information). Separation of THS–PAMAM from excess of PAMAM was carried out by size exclusion chromatography (HiPrep 16/60 Sephacryl S‐200 column) in SEC buffer (20 × 10^−3^ m Tris/HCl pH 7.5, 100 × 10^−3^ m NaCl, 1 × 10^−3^ m EDTA, 0.02% NaN_3_). Chromatograms at 280 and 350 nm were recorded and the peaks were pooled and concentrated. NaN_3_ was removed and buffer was exchanged to the buffers needed for the various further experiments by centrifugal filtration. All conjugation products were purified with Amicon centrifugal filters (THS: MWCO: 100 kDa; PAMAM: MWCO: 3 kDa) by at least five concentration–dilution cycles. Buffer exchange and concentration steps were carried out by centrifugal filtration in the same way.

*Molecular Substitution Ratio (MSR) of THS–MTFB*: To determine the degree of modification of THS with MTFB, THS–MTFB was reacted with 2‐hydrzinopyridine. 1.7 × 10^−6^ m THS–MTFB was reacted with 450 × 10^−6^ m 2‐hydrazinopyridine in 100 × 10^−3^ m sodium citrate buffer pH 5.0 for 30 min at 37 °C. The absorbance was measured at 350 nm, whereas the absorbance of the negative control (450 × 10^−6^ m 2‐hydrazinopyridine) was subtracted.

*MSR of PAMAM–HyNic*: PAMAM was modified with HyNic as described above, but the excess of HyNic over PAMAM was varied between 6 and 100. To measure the MSR, 10 × 10^−6^ m PAMAM–HyNic was incubated with 450 × 10^−6^ m 4‐nitrobenzaldehyde in buffer A for 30 min at 37 °C. The absorbance was measured at 390 nm (ε_390 nm_ = 24 000 M^−1^ cm^−1^)[[qv: 11a]] using a SpectraMax M5^e^ (Molecular Device) spectrometer, and the absorbance of the negative control (450 × 10^−6^ m 4‐nitrobenzaldehyde) was subtracted. The degree of modification could be controlled by the concentration of HyNic in the reaction mixture (Figure S1, Supporting Information).

*Characterization of THS–PAMAM Conjugates*: For SDS‐PAGE analysis of the conjugate, PAMAM–HyNic was labeled with Atto647‐*N*‐Hydroxysuccinimide (—NHS) by reacting PAMAM–HyNic (0.19 μg, 133 × 10^−6^ m) with a fivefold excess of Atto647–NHS (54.0 μg, 666 × 10^−6^ m) in buffer A for 1.5 h at RT in the dark. Unbound Atto647 was separated by dialysis (MWCO: 10 kDa) against 0.1 m NaCl (exchanged after 2 h, 4 h, and 8 h). Then, the sample was concentrated and the buffer exchanged as described above. Some of Atto647–PAMAM–HyNic was further linked to THS‐FB.

*Preparation of THS–Atto647*: Cysteines of THS were labelled with Atto647‐maleimide (Atto647‐mal) by reacting THS with a 40‐fold excess of Atto647‐mal. To this end, 7 × 10^−6^ m THS was reacted with 280 × 10^−6^ m Atto647‐mal in buffer B (100 × 10^−3^ m sodium phosphate pH 6.5, 150 × 10^−3^ m NaCl) for 2.5 h at RT under shaking. THS–Atto647 was purified from free dye with HiTrap desalting columns (Sephadex G25 Superfine; GE Healthcare, UK). Concentration and buffer exchange was performed with centrifugal filters (MWCO: 100 kDa).

*Preparation of THS–AuNP*: 2.3 × 10^−6^ m THS was reacted with a 20‐fold excess of 1.4 nm monomaleimido nanogold (AuNP‐mal) (46 × 10^−6^ m) in buffer B for 2 h at RT under gentle shaking. The sample was purified by size exclusion from unreacted AuNP by SEC as described in the method part of the paper (THS–PAMAM conjugation).

*Preparation of THS–PAMAM–AuNP*: 10 × 10^−6^ m PAMAM was modified with 30 × 10^−6^ m 1.4 nm mono‐sulfo‐NHS‐nanogold (AuNP–NHS) and 100 × 10^−6^ m HyNic in buffer A for 1.5 h at RT under gentle shaking. Free HyNic was removed with centrifugal filters (MWCO: 3 kDa). THS–MTFB was modified with AuNP–PAMAM–HyNic as described in the paper for the modification of THS–MTFB with PAMAM–HyNic.

*siRNA Binding to THS–PAMAM*: THS–PAMAM (3 × 10^−6^ M) was incubated with a fivefold excess of scrambled siRNA (21.0 μg, 15 × 10^−6^ m) in RNase‐free buffer A for 10 min at 37 °C. Unbound siRNA was removed by extensive centrifugal filtration (MWCO: 100 kDa). siRNA on the THS–PAMAM was quantified by UV/Vis spectroscopy. To this end, the absorbance spectrum of THS–PAMAM in the absence of siRNA was normalized to the absorbance of THS–PAMAM–siRNA at 354 nm. The concentration of siRNA was calculated from the difference in absorbance at 260 nm.

*Preparation of TAT–THS–PAMAM*: THS–PAMAM (5.4 × 10^−6^ m) was modified with a 20‐fold excess of tetra ethylene glycol pentafluorophenyl ester 4‐formylbenzamide (PEG_4_‐PFB) (**4**, 6.1 μg, 108 × 10^−6^ m) in buffer A for 2 h at RT under shaking. The product was purified and the buffer was exchanged to buffer B. The resulting PFB–THS–PAMAM (5.4 × 10^−6^ m) was reacted with a 20‐fold excess of TAT–HyNic (**5**, 20.0 μg, 108 × 10^−6^ m) in buffer B overnight at RT under shaking. Free TAT–HyNic was separated from TAT–THS–PAMAM by filter centrifugation (MWCO: 100 kDa).

*Cell Cultures*: All cell lines were grown at 37 °C under a 5% CO_2_ atmosphere in the following cell culture media (further labeled as “normal cell growth conditions”): HeLa & PC‐3: Dulbecco's modified Eagle medium (DMEM); CHO‐K1: Roswell Park Memorial Institute medium 1640 (RPMI); HUVEC: DMEM, 20 U mL^−1^ Heparin, 30 μL mL^−1^ endothelial cell growth supplement (ECGS); MCF‐7 and U‐87 MG: minimum essential medium Eagle (MEME) with nonessential amino acids (NEAA). All media were with 10% fetal calf serum (FCS), 200 U mL^−1^ penicillin and 200 μg mL^−1^ streptomycin. When cells reached a confluency of about 80%, they were split and subcultured by trypsinization.

*Live Cell Images*: For live images 50 000 cells were seeded in eight‐well microscope chamber slides for 24 h in normal cell culture conditions. Before adding the samples to the cells, old medium was removed; new medium was added to such an amount that in the end the total volume with sample resulted to be 100 μL. After 10 min or 2 h incubation under normal cell growing conditions the cells were washed with phosphate buffered saline (PBS) and 400 μL serum‐free medium was added. To stain nuclei, cell membrane and/or acidic compartments, the cells were incubated in 0.2 μg mL^−1^ Hoechst 33342 for 25 min and 2.5 μg mL^−1^ CellMask orange for 5 min or 20 × 10^−9^ m LysoTracker Red for 10 min, respectively. Subsequently, cells were washed with PBS and wells were refilled with 400 μL PBS. Cell imaging was performed by confocal laser scanning microscopy (CLSM) with a Zeiss 510‐META/Confocor2 microscope equipped with a laser diode (405 nm), argon laser (488 nm), and a helium/neon laser (633 nm); and a 40× water‐immersion objective (Zeiss C‐Apochromat 40×/1.2 W corr). The samples were excited at 405 nm, 514 nm (NFT mirror 545), or 633 nm (NFT mirror 545) and the emission was collected with a broad pass filter at 420–480 nm, a broad pass filter 560–615 nm or an long pass filter at 650 nm, respectively. The pinholes were set to 61, 80, and 92 μm, respectively. The cells were imaged in multitrack mode with a resolution of 1024 × 1024 pixels (225 μm × 225 μm) with a pixel time of 25.6 μs. The images were cropped to 512 × 512 pixels (112 μm × 112 μm) using LSM Image Browser (Zeiss).

*FACS*: For FACS measurements, 100 000 cells were seeded into 24‐well plates, filled up with 1.5 mL cell culturing medium and incubated under normal cell culture conditions. After 2 d, the medium was aspirated, THS–Atto647 or TAT–THS–Atto647 were added and filled up to 100 μL with cell culturing medium so that the final dye concentration was 150 × 10^−9^ m. After 2 h of incubating under normal cell grow conditions, the cells were washed with PBS, trypsinized, and put on ice. FACS measurements were performed with a FACSCanto II (BD, USA) with at least 10 000 cells. The cells were excited with a HeNe laser at 633 nm and the emitted light passed through a Band Pass 660/20 filter before reaching the detector. Data were processed with FlowJo Vx (Tree Star, USA) and a histogram of fluorescence of single cells only was plotted.

*Dynamic Light Scattering*: DLS measurements of 0.1 × 10^−6^ m THS–PAMAM, and 0.1 × 10^−6^ m THS–PAMAM incubated for 10 min at RT with 0.1 × 10^−6^ m siRNA in PBS pH 7.4 were performed on a Zetasizer ZSP (Malvern, USA) instrument. The backscattering at 173° was recorded at 25 °C with an equilibrium time of 30 s. The data were analyzed with the instrument's software. Each experiment was carried out four times. The reported data represents the average of these four measurements, based on particle number distributions. All samples were measured six times.

*KIF11 siRNA Delivery*: Silencer KIF11 (Eg5) siRNA was used as active siRNA and scrambled siRNA was used as negative control. KIF11 siRNA or scrambled RNA was bound to THS–PAMAM or TAT–THS–PAMAM as described above. siRNA transfection was performed according to the reversed siRNA transfection protocol from the manufacturer of LF (Life Technologies, USA). For this purpose THS–PAMAM–siRNA, TAT‐THS–PAMAM–siRNA, PAMAM–siRNA (in a ratio of 3.8 eq. PAMAM to 1 eq. siRNA, which corresponds to the ratio in THS–PAMAM–siRNA), 0.3 μL Lipofectamine RNAiMAX and siRNA, or 0.5 μL LF and siRNA were pipetted into wells of a 96‐well plate and filled up to 10 μL with RNase free buffer A (100 × 10^−3^ m sodium phosphate pH 7.5, 150 × 10^−3^ m NaCl) so that a siRNA concentration of 1 × 10^−6^ m was achieved. After 10 min, 5000 cells were added to each well and filled up to 100 μL with medium without serum and antibiotics, resulting in a final siRNA concentration of 100 × 10^−9^ m. This corresponds to 10 pmol of siRNA per well. All experiments were conducted with KIF11 siRNA and scrambled siRNA to calculate the viability decrease due to KIF11 siRNA. The medium was exchanged to normal growth medium after 5 h. The cells were incubated 72 h in total under normal growth conditions. The cell viability was determined with the cell counting kit‐8 (CCK‐8; Sigma‐Aldrich). Absorbance of converted CCK‐8 was measured at 450 nm with a SpectraMax M5^e^ plate reader (Molecular Devices). As background CCK‐8 in medium was measured, which was subtracted from the data. The viabilities of cells treated with siRNA and scrambled RNA were normalized to the viability of untreated cells. All measurements were performed in multiple, whereas *n* indicates the number of experiments. All data were first tested with Prism (GraphPad, USA) to confirm that they are normally distributed and have the same variance. As this was the case, two‐sided Student's t‐test was applied, whereas significance level was set to *p* < 0.05 (*), *p* < 0.01 (**) and *p* < 0.0001 (****). Data are presented as mean ± SD.

*GAPDH siRNA Delivery*: The procedure of a previously published protocol was followed with several modifications.^24^ 24 h before the experiment, 5000 U87 cells were plated in 100 μL per well in a 96‐well plate and incubated at 5% CO_2_ and 37 °C. For every sample, four wells were plated. After 24 h, samples were prepared by adding 0.4–40 pmol of GAPDH siRNA (volume 20 μL) in opti‐MEM, and mixing it with either blank opti‐MEM, opti‐MEM containing Lipofectamine RMAiMAX (0.3 μL per pmol of siRNA), THS–PAMAM (in a ratio of 4:1 siRNA to THS–PAMAM) or PAMAM (in a ratio of 4:1 siRNA to PAMAM) in 20 μL (total volume 40 μL). The samples were incubated at room temperature for 30 min. 10 μL of sample were added to each well (final concentrations of 0.1–10 pmol siRNA per well). After 72 h, the media was removed from cells and they were washed three times with PBS. 60 μL of lysis buffer was added to the first well, which was incubated for 5 min and then transferred to the second, third, and fourth well of each sample with 5 min incubation in each (final volume of 60 μL containing a mixture of the cell lysate of all four wells, thus physically averaging the results of four experiments). The protein concentration of the cell lysate was quantified with the micro‐BCA protein assay kit. 15 μg total protein of each sample was then mixed in 5× SDS protein loading buffer and heated at 95 °C for 10 min. After heating, the samples were loaded into a 10% TGX stain free acrylamide gel and electrophoresed at 120 V for 50 min. The gel was removed from the cassette and imaged on an EZdoc one touch imager (BioRAD) using the TGX stain free method activating for 5 min. This image was analyzed in ImageJ to verify consistent loading of each well by quantifying the band at 40 kDa (see Figure S11, Supporting Information for the loading control gels that correspond to the Western blots of the manuscript). The gels were then transferred to nitrocellulose membranes via a biometra FASTblot semidry transfer system at a constant 80 mA for 25 min. The membranes were blocked in 5% milk in TBS buffer overnight at 40 °C. The next morning the blocking solution was removed. 15 mL of 5% milk in TBS containing 1:10 000 mixture of primary monoclonal mouse antiGAPDH antibody was added to each membrane. They were incubated at room temperature for 2 h. The membranes were then washed three times in TBST buffer for 10 min. The second incubation was done in 15 mL 5% milk in TBS containing 1:10 000 goat antimouse HRP conjugated antibody for 1 h. The membranes were washed three more times at 10 min each in TBST buffer and then dried. Supersignal west pico chemiluminescent substrate was added as per manufacturer's directions and imaged on a Bucher biotec fluorescence imaging system equipped with s FujiFilm Las‐4000 camera. Images were recorded with the Las‐4000 software and analyzed in ImageJ to determine the GAPDH‐protein level of each sample.

## Supporting information

As a service to our authors and readers, this journal provides supporting information supplied by the authors. Such materials are peer reviewed and may be re‐organized for online delivery, but are not copy‐edited or typeset. Technical support issues arising from supporting information (other than missing files) should be addressed to the authors.

Supplementary
