# Supplementary material for: Chaperonin–Dendrimer Conjugates for siRNA Delivery
Source: Adv Sci (Weinh). 2016 May 27;3(10):1600046. doi: 10.1002/advs.201600046 (PMC5096033; doi:10.1002/advs.201600046)
Supplement: Supplementary file 1 — Supplementary [file ADVS-3-0s-s001.pdf]

Copyright WILEY-VCH Verlag GmbH & Co. KGaA, 69469 Weinheim, Germany, 2016.

ADVANCED SCIENCE
Open Access

Supporting Information

for *Adv. Sci.*, DOI: 10.1002/advs.201600046

Chaperonin–Dendrimer Conjugates for siRNA Delivery

*Martin G. Nussbaumer, Jason T. Duskey, Martin Rother, Kasper Renggli, Mohamed Chami, and Nico Bruns**

Copyright WILEY-VCH Verlag GmbH & Co. KGaA, 69469 Weinheim, Germany, 2016.

Supporting Information

Chaperonin-Dendrimer Conjugates for siRNA Delivery

*Martin G. Nussbaumer, Jason. T. Duskey, Martin Rother, Kasper Renggli, Mohamed Chami and Nico Bruns**

Dr. M. G. Nussbaumer, Dr. J. T. Duskey, Dr. M. Rother, Dr. K. Renggli, Dr. N. Bruns
Department of Chemistry, University of Basel, Klingelbergstrasse 80, 4056 Basel, Switzerland

Dr. M. Chami
C-CINA, Center for Cellular Imaging and NanoAnalytics, Biozentrum, University of Basel, Mattenstrasse 26, 4058 Basel, Switzerland

Prof. N. Bruns
Adolphe Merkle Institute, University of Fribourg, Chemin des Verdiers 4, 1700 Fribourg, Switzerland
E-mail: nico.bruns@unifr.ch

Current addresses:
Dr. M. G. Nussbaumer
Wyss Institute for Biologically Inspired Engineering
Harvard University
Cambridge, MA 02138, USA

Dr. K. Renggli
Department of Biological Engineering, Massachusetts Institute of Technology, 77 Massachusetts Avenue, Cambridge, MA 02139, USA

Table S1: UV absorbance measurement of THS-MTFB reacted with 2-hydrazinopyridine to calculate the molecular substitution ratio (MSR) of THS with the linker MTFB.

Wavelength [nm]	Absorbance	ε [M^{-1} cm^{-1}]	c [µM]	MSR
280	0.036	210880[1]	1.7	3.8
350	0.012	18000[1]	6.5	

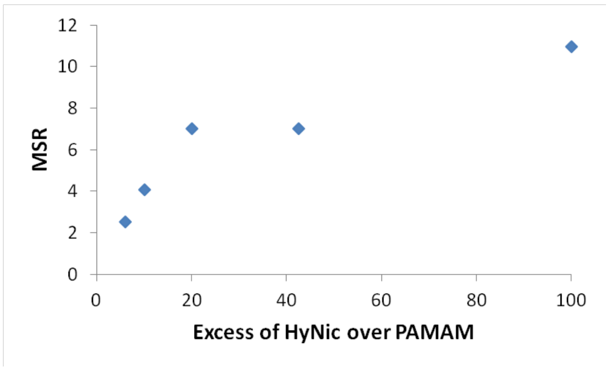

Figure S1. Controlling the degree of modification of PAMAM with HyNic by varying the excess of HyNic over PAMAM.

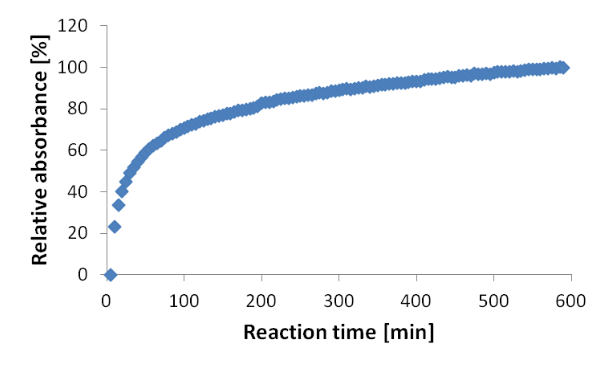

Figure S2. Kinetics of THS-PAMAM formation. The UV/Vis absorbance at 350 nm during the reaction of THS-MTFB with PAMAM-HyNic was measured every 5 min over 10 h using a SpectraMax M5e (Molecular Device) spectrometer. The absorbance was normalized to the absorbance at a reaction time of 10 h.

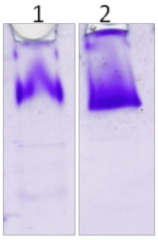

Figure S3. Native PAGE of THS (1) and THS-PAMAM (2).

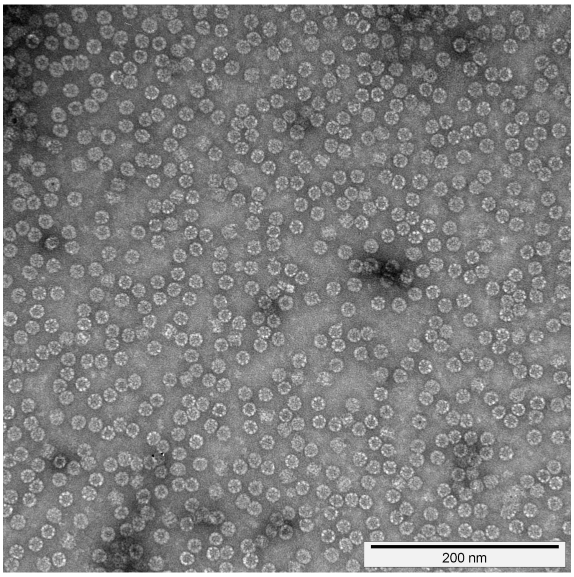

Figure S4. TEM image of THS-PAMAM conjugate.

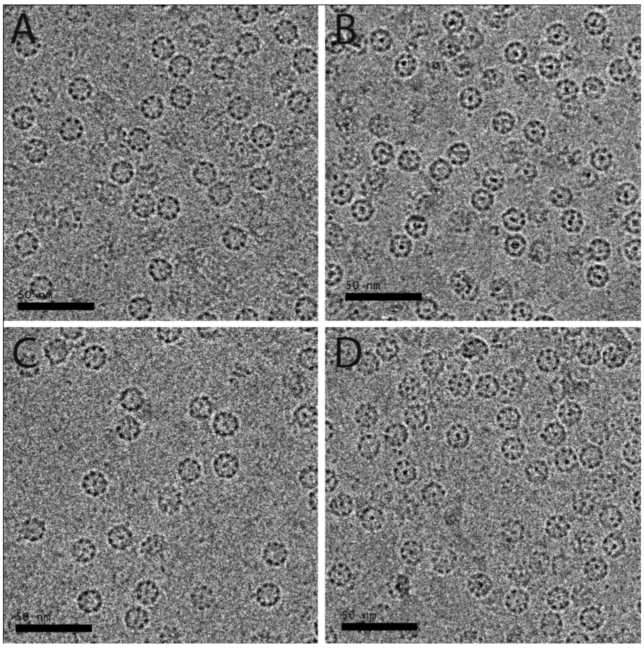

Figure S5. Cryo-TEM images of THS (A), THS-AuNP (B), THS-PAMAM (C) and THS-PAMAM-AuNP (D). The cryo-TEM images shown in **Figure 3b** have been cropped from A and D and are discussed in the manuscript. Micrograph A shows the structure of THS, and micrograph D shows that AuNP-labelled PAMAM was located within the THS. A cryo-TEM image of THS-PAMAM (C) is presented here for comparison. In addition, THS was labelled with AuNP-maleimide to locate the cysteines of the engineered protein cage (B). Several AuNP per THS were found inside THS, confirming the location of cysteines within the protein cage's cavities. Scale bars are 50 nm.

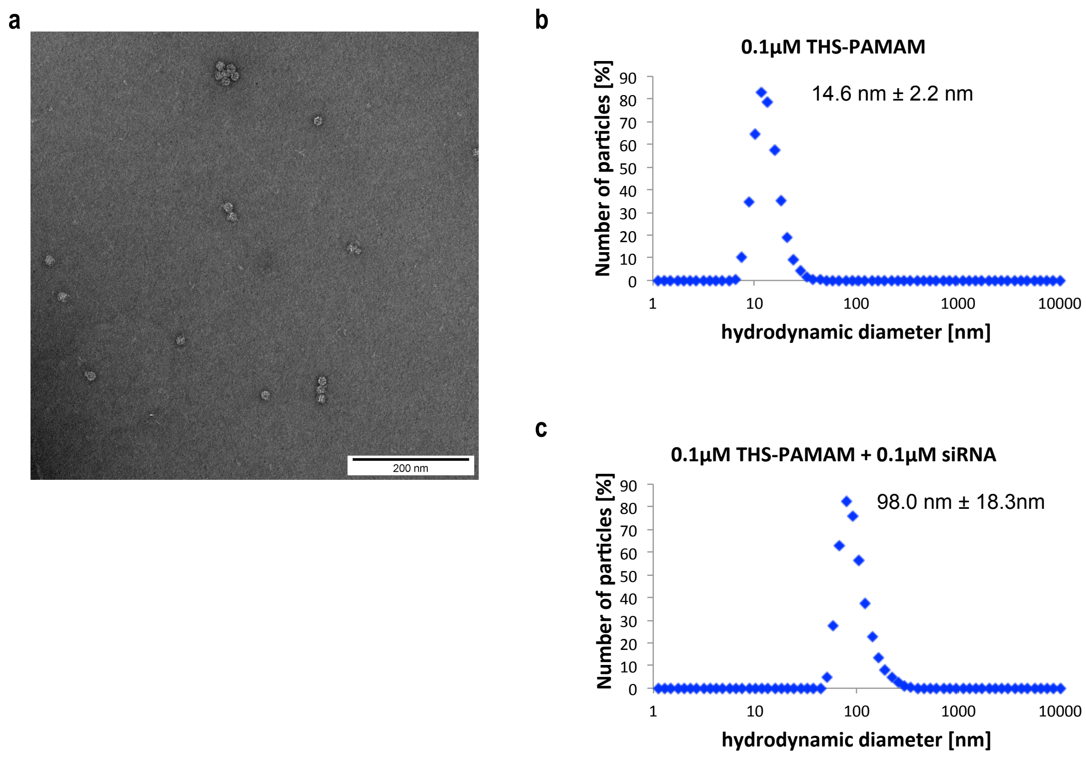

Figure S6. Morphology of siRNA/THS-PAMAM complexes and of siRNA/PAMAM complexes. a) TEM of 1 nM THS-PAMAM with 1 nM siRNA. b) DLS measurements of 0.1 µM THS-PAMAM. c) DLS measurements of 0.1 µM THS-PAMAM with 0.1 µM siRNA.

Table S2. Cell uptake of THS-Atto647: Quantification of FACS results presented in Figure 6a and comparison to FACS results obtained with free dye. Increase of median fluorescence for different cell lines incubated with THS-Atto647 and Atto647, respectively, over background cell fluorescence. Values: n-fold increase (robust coefficient of variation).

	THS-Atto647	Atto647
HeLa	5.3 (67%)	3.5 (57%)
PC-3	3.0 (58%)	2.3 (51%)
MCF-7	1.7 (74%)	12.1 (86%)
U-87 MG	46.8 (59%)	29.5 (52%)
CHO-K1	1.9 (50%)	n.d.
HUVEC	3.2 (103%)	n.d.

n.d. = not determined

The results show that THS interacted strongly with U87 cells, but not with the other cell lines that were tested.

The comparison between THS-Atto647 and free dye is instructive, because it reveals a common pitfall when measuring dye-cell interactions by FACS. A problem of many uptake studies that use fluorescent labels is that the dyes are located on the surface of particles so that they can interact with cells. Furthermore, dyes often promote cellular internalization due to their cationic or hydrophobic properties, resulting in an alleged uptake signal.[2] The THS overcomes this problem, because the fluorophore is conjugated to cysteines that are located inside of the THS's cavities. HeLa and PC-3 cells incubated with Atto647 exhibit a similar median fluorescence as when incubated with THS-Atto647, indicating that Atto647 does not interact with these cells. However, fluorescence intensity of MCF-7 cells was much higher when incubated

with Atto647 than with the fluorescently labeled THS, suggesting that the free dye bound to the cells, while the protein cage shielded Atto647 from direct interactions with cells. U-87 MG cells also showed an enhanced fluorescence by the free dye. However, a more efficient binding or uptake of THS-Atto647 was observed. From these results, we infer that U-87 MG could be targeted with THS for siRNA delivery.

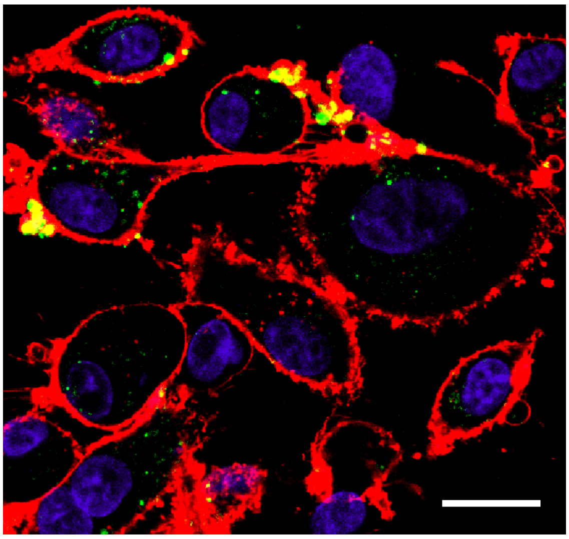

Figure S7. Confocal fluorescence image of U-87 MG cells incubated with Atto647 for 2 h. Atto647 (green), nucleus (blue), cell membrane (red); scale bar: 20 µm.

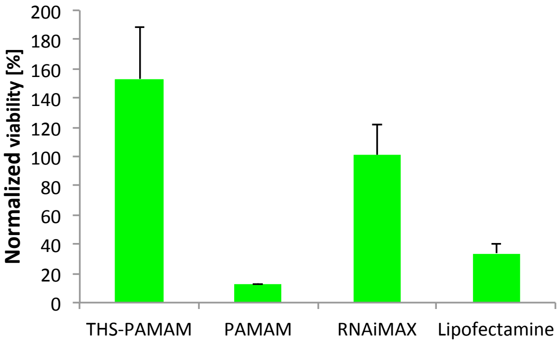

Figure S8. Normalized viability of U-87 MG cells incubated with THS-PAMAM, PAMAM, Lipofectamine® RNAiMAX or Lipofectamine® 2000 at the same concentrations and conditions used in the siRNA delivery assay, but without siRNA.

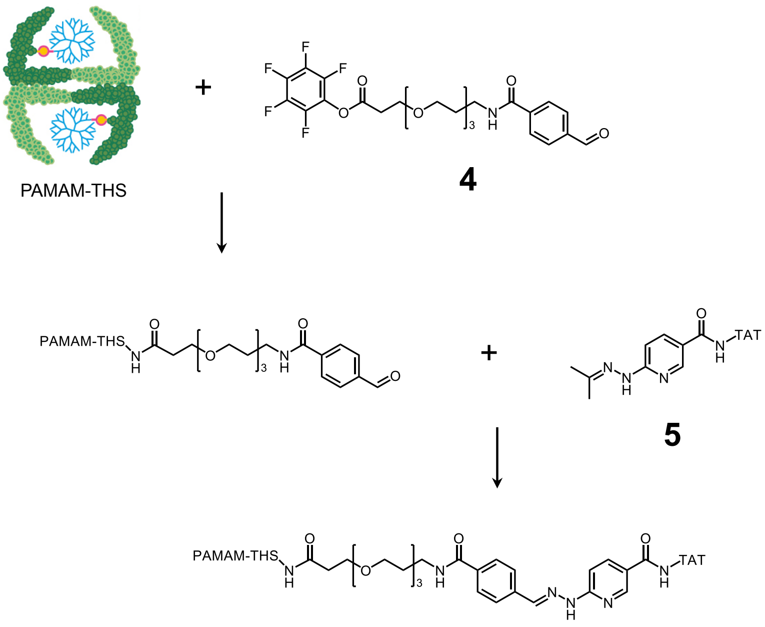

Figure S9. Strategy to conjugate TAT-HyNic to THS-PAMAM. Lysines of THS-PAMAM were modified with the heterobifunctional linker PEG4-PFB (4). After separation of free linker, PFB-THS-PAMAM and TAT-HyNic (5) were reacted to TAT-THS-PAMAM.

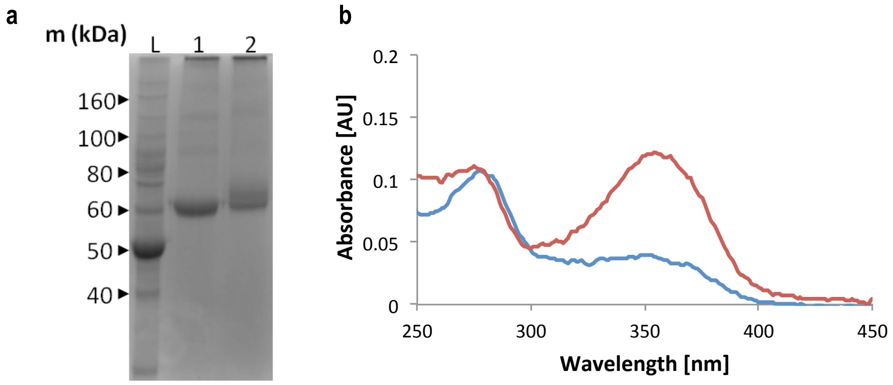

Figure S10. Modification of THS-PAMAM with TAT. a) SDS-PAGE of THS-PAMAM (1) and TAT-THS-PAMAM (2). b) UV/Vis spectrum of THS-PAMAM (blue) and TAT-THS-PAMAM (red). The increase of the bis-arylhydrazone absorption band at 354 nm proves the formation of linkers between TAT and THS-PAMAM and was used to quantify the number of TAT per THS.

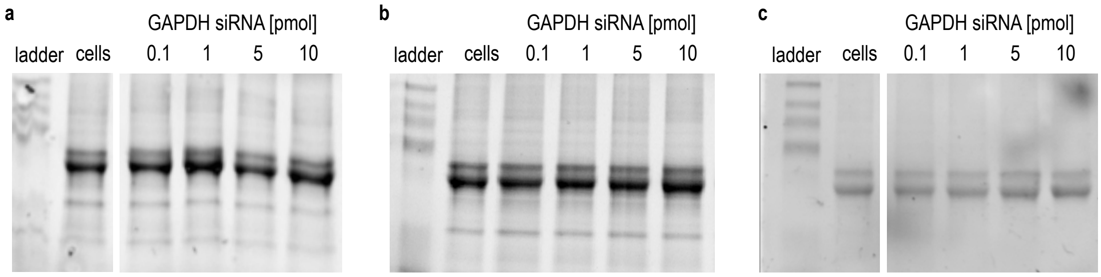

Figure S11. siRNA delivery into U87 cells. Loading control gels for western blots presented in Figure 7. a) THS-PAMAM, b) PAMAM, and c) RNAiMAX.

[1] N. Bruns, K. Pustelny, Lisa M. Bergeron, Timothy A. Whitehead, Douglas S. Clark, *Angew. Chem. Int. Ed.,* **2009**, *48*, 5666.

[2] a) F. Heitz, M. C. Morris, G. Divita, *Br. J. Pharmacol.,* **2009**, *157*, 195; b) A. T. Jones, E. J. Sayers, *J. Control. Release,* **2012**, *161*, 582.
